# Deciphering the Mechanism of *β*-Aminobutyric Acid-Induced Resistance in Wheat to the Grain Aphid, *Sitobion avenae*

**DOI:** 10.1371/journal.pone.0091768

**Published:** 2014-03-20

**Authors:** He-He Cao, Meng Zhang, Hui Zhao, Yi Zhang, Xing-Xing Wang, Shan-Shan Guo, Zhan-Feng Zhang, Tong-Xian Liu

**Affiliations:** State Key Laboratory of Crop Stress Biology for Arid Areas, and the Key Laboratory of Crop Pest Management on the Losses Plateau of Ministry of Agriculture, Northwest A&F University, Yangling, Shaanxi, China; Zhejiang University, China

## Abstract

The non-protein amino acid *β*-aminobutyric acid (BABA) can induce plant resistance to a broad spectrum of biotic and abiotic stresses. However, BABA-induced plant resistance to insects is less well-studied, especially its underlying mechanism. In this research, we applied BABA to wheat seedlings and tested its effects on *Sitobion avenae* (F.). When applied as a soil drench, BABA significantly reduced weights of *S. avenae*, whereas foliar spray and seed treatment had no such effects. BABA-mediated suppression of *S. avenae* growth was dose dependent and lasted at least for 7 days. The aminobutyric acid concentration in phloem sap of BABA-treated plants was higher and increased with BABA concentrations applied. Moreover, after 10 days of treatment, the aminobutyric acid content in BABA-treated plants was still higher than that in control treatment. *Sitobion avenae* could not discriminate artificial diet containing BABA from standard diet, indicating that BABA itself is not a deterrent to this aphid. Also *S. avenae* did not show preference for control plants or BABA-treated plants. Consistent with choice test results, *S. avenae* had similar feeding activities on control and BABA-treated plants, suggesting that BABA did not induce antifeedants in wheat seedlings. In addition, aminobutyric acid concentration in *S. avenae* feeding on BABA-treated plants was significantly higher than those feeding on control plants. *Sitobion avenae* growth rate was reduced on the artificial diet containing BABA, indicating that BABA had direct toxic effects on this aphid species. These results suggest that BABA application reduced *S. avenae* performance on wheat seedlings and the mechanism is possibly due to direct toxicity of high BABA contents in plant phloem.

## Introduction

The non-protein amino acid *β*-aminobutyric acid (BABA) can enhance plant resistance against a broad spectrum of phytopathogens. BABA-induced plant resistance is effective against viruses, bacteria, oomycetes, fungi and phytopathogenic nematodes [1]–[4]. The mechanism of BABA-induced plant resistance to different pathogens is variable. BABA-induced *Arabidopsis* resistance against the oomycete pathogen *Peronospora parasitica* (Pers.) Fr. is based on callose deposition, and independent of jasmonic acid (JA), salicylic acid (SA) and ethylene signaling pathways, whereas against the bacteria *Pseudomonas syringae* is solely dependent of SA and NPR1 (nonexpressor of pathogenesis-related genes 1) [3]. Enhanced resistance of tobacco to tobacco mosaic virus by BABA application was found to be strictly dependent on SA pathway [2]. BABA-mediated grapevine resistance to downy mildew (*Plasmopara viticola* Berk. & M.A. Curtis) is based on potentiating callose formation and JA signaling [5]. In addition to conferring plant resistance to pathogens, BABA also improves plant tolerance to salt, drought and high temperature, which is associated with accumulation of the abscisic acid [6]–[8]. *Arabidopsis* mutants that are insensitive to BABA-induced sterility have reduced resistance level to pathogens or tolerance to salt, suggesting that BABA-induced plant resistance to biotic and abiotic stresses has a genetic basis [9].

BABA has been demonstrated to be effective in plant resistance to insects [10]–[12]. Hodge et al. (2005) found that BABA applied as a root drench to six legume plant species reduced the performance of the pea aphid, *Acyrthosiphon pisum* (Harris) [10], which was the first study that examined the effects of BABA-mediated plant resistance to insects. When applied to Brassicaceae plants, BABA suppressed the growth of phloem-feeding insects *Myzus persicae* (Sulzer) and *Brevicoryne brassicae* (L.) as well as chewing insects *Trichoplusia ni* (Hübner) and *Plutella xylostella* (L.) [11]. Recently, Tiwari et ) reported that BABA application also induce citrus resistance to the Asian citrus psyllid, *Diaphorina citri* Kuwayama, and the authors did not find any direct toxicity of BABA to this insect by leaf-dipping bioassays [12]. Although these studies indicate that BABA can enhance plant resistance to insects, little is known about the underlying mechanism.

Plant direct resistance to insects relies largely on metabolites that exert toxic, antinutritive, or repellent effects. Insects could perceive most of these compounds by chemoreceptors and decide to accept or reject a host [13]. The phloem-feeding insects, like aphids and whiteflies, have evolved specialized mouthparts, the stylets, which penetrate the plant epidermis, pass through the intercellular tissue, and search for sieve element. During stylet penetration, aphids regularly puncture plant cells and ingest cytosolic contents to decide to feed or leave before reaching phloem [14]. Once they have reached the sieve element, aphids will always eject watery saliva to prevent sieve tube plugging before phloem sap ingestion. The probing behavior of aphids can be monitored by the electrical penetration graph (EPG) technique [15].

The English grain aphid, *Sitobion avenae* (F.), is a major pest of cereal crops worldwide [16]. This aphid causes substantial losses of cereal yield by removing photoassimilates and transmitting viruses. Application of chemical pesticides is still the main method to control aphids; however, chemical control causes negative impacts on agroecosystems and can lead to insect resistance to pesticides [16]. Thus searching for alternative methods to control this aphid is of great significance.

Understanding the mechanism of BABA-induced plant resistance to insects will shed light on new methods of pest control. In this study we examined the effects of BABA and its isomers α-aminobutyric acid (AABA) and γ-aminobutyric acid (GABA) application to wheat on performance of the *S. avenae*. The durability of BABA-induced resistance was also investigated. Host preference and feeding behavior of *S. avenae* were studied to localize possible resistance factors. We found that BABA accumulated to a high concentration in BABA-treated wheat phloem sap; therefore, we added BABA to aphid artificial diet to test its direct toxicity to *S. avenae*.

## Materials and Methods

### Plants and insects

Seeds of winter wheat, *Triticum aestivum* (var. ‘XiNong 979’), were germinated at room temperature (25±1°C) for 2 days. Seedlings were then transplanted to pots (250 mL) containing a 5∶1 mixture of peat moss (Pindstrup Mosebrug A/S, Ryomgaard, Denmark) and perlite. Seedlings were grown singly in pots unless otherwise indicated. Plants were cultivated in a walk-in growth chamber at 24±1°C, 60±5% relative humidity, and a 14∶10 h light/dark regime. The plants were watered as necessary. *Sitobion avenae*, originally collected from a winter wheat field in Yangling, China, was reared on wheat seedlings (var. ‘XiNong 979’) in the same growth chamber.

### *Sitobion avenae* performance on wheat seedlings

For soil drench application, we poured 20 mL of each treatment solution (dissolved in MilliQ water) to the soil of each pot and the control solution was MilliQ water. The foliar treatments were made by spraying control solution (MilliQ water containing 0.05% Triton X-100) or 50 mM BABA (dissolved in control solution) until runoff with a 30 mL hand sprayer. For seed treatments, 60–100 wheat seeds were incubated in a 4°C refrigerator in MilliQ water or 50 mM BABA for 24 h. Seeds were then washed six times in MilliQ water before sowing. Seven-day-old wheat seedlings were treated as described and then 2–3 apterous *S. avenae* adults were introduced to the first fully expanded leaf of each seedling. The seedlings were individually caged in transparent plastic cages (8 cm in diameter and 30 cm in height) covered with nylon mesh at the top. One day later, the adults were removed, leaving 5–6 nymphs on the first leaf. After another 7 days, all aphids on the first leaves were collected and weighed on a microbalance (resolution 0.001 mg; Sartorius MSA 3.6P-000-DM, Gottingen, Germany). To assess the effects of BABA treatment on plant growth, the fresh shoot weights of wheat seedlings were weighed after aphid collection. Weights of fresh wheat shoots and aphids feeding on wheat seedlings soil-drenched with MilliQ water, different concentrations of BABA and 50 mM GABA were analyzed using one-way analysis of variance (one-way ANOVA) following by Tukey's HSD (Honestly Significant Difference) test. Mean weights of *S. avenae* feeding on plants soil-drenched with 50 mM AABA, sprayed with BABA, or seed treatment with BABA were compared with respective control by Student's *t*-test. Wheat seedlings weights were analyzed using one-way ANOVA following by Tukey's HSD test. All statistical analyses in this research were conducted using IBM SPSS Statistics package (version 19.0; SPSS Inc., Chicago, IL, USA).

### *Sitobion avenae* settling choices

Seven-day-old wheat seedlings were soil-drenched with 20 mL MilliQ water (control) or 25 mM BABA. One day after treatment, one control and one BABA-treated seedling were placed in a transparent plastic cage (26 cm height ×15 cm length ×14 cm width) covered with nylon mesh at the top. Fifteen alate adults of *S. avenae* were introduced to each cage, and numbers of *S. avenae* on each seedling were recorded after 4, 8, 24 and 48 h. Because we found that alate *S. avenae* adults showed a strong preference to light and usually flied to the top of cages, we performed this assay in a completely dark room to prevent aphid taking off from plants. Eight replicates were performed. Numbers of aphids on control and BABA-treated plants at each time point were compared with a paired *t*-test.

To determine whether BABA itself has direct antifeedant effects, we tested *S. avenae* preference to artificial diet containing BABA. The composition of standard artificial diet was based on Auclair (1965) [17] with modifications (Table S1). After sterile filtering through Millex GP syringe filters (hydrophilic polyethersulfone membrane, pore size 0.22 μm; Ireland), the artificial diets were stored at −70°C until use. The Petri dish lid (7 mm in height and 37 mm in diameter) was covered with a layer of stretched Parafilm M (Chicago, IL). Forty μL of MilliQ water (H_2_O), artificial diet (Control), and artificial diet containing 50 mM BABA were put on the Parafilm M separately. The test solutions were then covered with another layer of Parafilm M. We made a hole (5 mm in diameter) on the back of each lid and introduced 21 3rd-5th instar aphids through this hole. The numbers of aphids feeding on each test solution were recorded after 6, 12, 24 and 48 h. Fourteen replicates were performed. Percentages of aphids on different test solutions within different recording time points were analyzed with one-way ANOVA, and means were compared using Tukey's HSD test.

### *Sitobion avenae* feeding behavior

Aphid feeding activities on control and BABA-treated plant seedlings were recorded by the Giga-8 direct-current electrical penetration graph (DC-EPG) system [18]. The detail of this experiment can be found in our previous work [19]. Wheat seedlings were soil-drenched with 20 mL MilliQ water (Control) or 25 mM BABA two days before EPG recording. Each apterous adult and each wheat seedling were used only once. Data were recorded by the Stylet+d software and analyzed with the Stylet+ software. The stylet pathway waveforms were distinguished according to Tjallingii (1978) [15]. EPG parameters were calculated using the Excel workbook for automatic parameter calculation of EPG data 4.3 [20]. Because the EPG data were not normally distributed, paired comparison of means of control treatment and BABA treatment was done by non-parametric Mann–Whitney *U*-test.

### Amino acid extraction and analysis

We used EDTA-facilitated exudation method to collect phloem sap of wheat seedling leaves for free amino acid analysis [21]. Wheat seedlings were soil-drenched as described 3 days before sample collection. The first leaf of each seedling was cut 1 cm above the leaf/stem junction and immediately placed in a 1.5 mL EP tube containing 600 μL EDTA solution (pH  = 7.1). Two leaves were placed in each EP tube and regarded as one replicate. Then these leaves were placed in a completely dark growth chamber at 25°C with 100% relative humidity for 3 hours. The samples were stored at −70°C until analysis.

Newly-born *S. avenae* nymphs feeding on control and BABA-treated plants for 7 days were collected and weighed. Free amino acids in aphids were extracted with 50% ethanol containing 0.1 M HCl. The free amino acids extracted from plants and aphids were analyzed by LTQ XL linear ion trap mass spectrometer (Thermo Scientific, Waltham, MA, USA). Liquid chromatography separations were carried out with XTerra MS C18 Column (125Å pore size, 5 μm, 150 mm ×4.6 mm; Waters Corp., Milford, MA, USA). Amino acid elution was performed applying a three-step gradient: A 100% for 7 min, 0–100% B linear for 2 min, 100% B for another 5 min, and 0–100% A linear for 1 min, holding the system at 100% A for 5 min. Mobile phase A was a aqueous solution containing 5% acetonitrile and 0.1% formic acid; and mobile phase B was 100% acetonitrile. The flow rate was 0.6 mL/min. The mass spectrometer worked in the positive electrospray ionization (ESI) mode. Nitrogen was used as the sheath gas (50.0 arbitrary units) and auxiliary gas (8.0 arbitrary units). The spray voltage was set at 4.5 kV and the ion transfer capillary temperature was 320°C. The amino acids were scanned and fragmented using data dependent MS/MS. Masses of precursor and product ions and collision energy for each amino acid were as described in Table S2. Data were acquired and processed using Xcalibur 2.1 software (Thermo Scientific, Waltham, MA, USA). Quantification was achieved by external standard amino acid mixture of known concentrations (AA-S-18, Sigma). Because our equipment cannot detect glycine, the dataset from this study did not include glycine. Mean concentrations of each amino acid from different treatments were compared by Student's *t-*test.

### Time course study

To investigate the durability of the effects of BABA treatment on *S. avenae* performance and phloem sap amino acid composition, we conducted a time course experiment. Seven-day-old wheat seedlings were soil-drenched with 20 mL MilliQ water or 25 mM BABA, and aphid performance assays started 0, 7 and 14 days after treatment; phloem sap was collected 3, 10 and 17 days after treatment for amino acid analysis. Aphid weights and aminobutyric acid concentrations in plant phloem sap of different treatments within each sample day were analyzed by Student's *t*-test.

### Peroxidase assays

Peroxidase (POD) is involved in some forms of plant resistance to insects [22]. The first leaves of control and BABA-treated plants were collected 2, 4 and 6 days after treatment, extracted and analyzed as described previously [19]. POD activities of control and BABA-treated plants within each sample day were compared by Student's *t*-test. In another experiment, plants were divided to following four treatments: (1) control plants (soil-drenched with water); (2) control plants infested with fifteen 3rd instar-adult *S. avenae*; (3) BABA-treated plants (soil-drenched with 25 mM BABA); (4) BABA-treated plants infested with fifteen 3rd instar-adult *S. avenae*. After 2 and 4 days of treatment, the first leaves were collected and analyzed as described. Mean activities of POD among treatments on each day were analyzed by one-way ANOVA, with Tukey's HSD post-hoc test.

### Artificial diet assays

We tested the direct impacts of BABA on performance of *S. avenae* by measuring nymphal growth on standard artificial diet and artificial diet containing 50 mM BABA, AABA, or GABA. We used AABA and GABA in artificial diets to exclude the possibility that the imbalance of artificial diet has negative impacts on aphid growth. Thirty-five μL of artificial diet was confined between two layers of stretched Parafilm M on plastic cylinder (1 cm in height and 1cm in diameter) and the artificial diet was replaced every two days. Five one-day-old nymphs produced by apterous *S. avenae* were transferred to one tube containing one kind of artificial diet and regarded as one replicate. The weight of individual nymphs was weighed on the MSA 3.6P-000-DM microbalance after feeding on artificial diet for 4 days. We also tested aphid performance on artificial diet containing 50 mM and 100 mM BABA in a similar manner, but the one day old nymphs used were produced by *S. avenae* collected from wheat seedlings (var. ‘XiNong 979’) in the greenhouse. Weights of aphids were analyzed with one-way ANOVA following by Tukey's HSD test.

## Results

### *Sitobion avenae* performance

**Figure 1:**
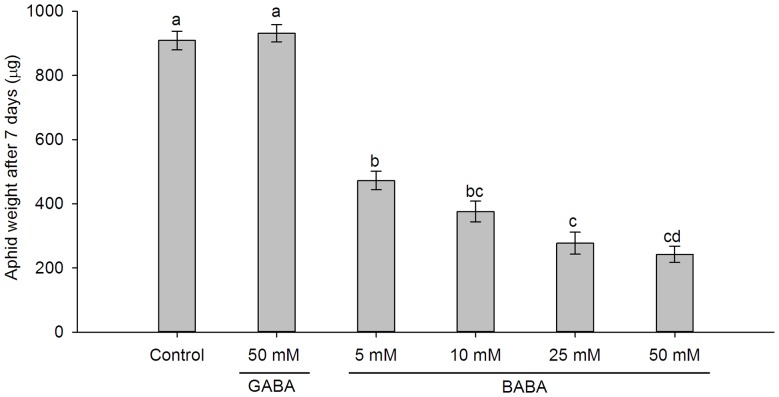
*Sitobion avenae* weights after feeding for 7 days on wheat seedlings with different treatments. Aphid weights on wheat plants soil-drenched with MilliQ water (control), 50 mM GABA, different concentrations of BABA. Bars represent mean ± SEM. Different letters above bars indicate statistically significant differences (*P*<0.05, Turkey's HSD test).

Compared with the control treatment, BABA applied as a soil drench significantly reduced weights of *S. avenae* on wheat seedlings and the effects increased with BABA concentration (*F* = 107.66, df  = 5, 24, *P*<0.001; Fig. 1). In contrast, soil drench with GABA (*P* = 0.994; Fig. 1) and AABA (*t* = 0.298, df  = 27.03, *P* = 0.768; data not shown), spray with BABA (*t* = 0.285, df  = 24, *P* = 0.778; data not shown), or seed treatment with BABA (*t* = 0.002, df  = 59, *P* = 0.999; data not shown) had no negative impacts on *S. avenae* weights. Wheat seedlings that soil-drenched with high concentration (50 mM) BABA had lower fresh shoot weights compared with the control treated-plants (*F* = 4.476, df  = 4, 44, *P* = 0.004) (Fig. S1).

### *Sitobion avenae* preference

**Figure 2:**
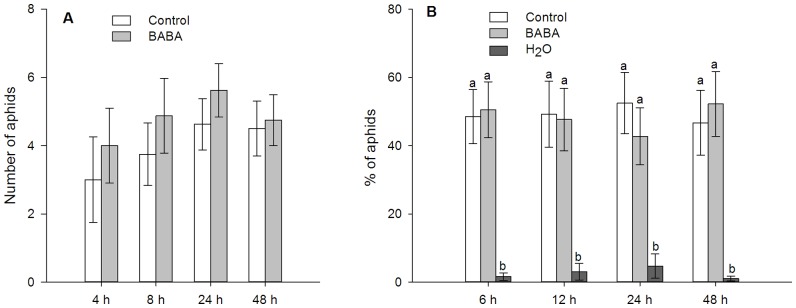
Settling preference of *Sitobion avenae*. (A) Mean number (± SEM) of *S. avenae* settling on control or BABA-treated plants (n = 8, paired *t*-test). Plants were soil-drenched with MilliQ water (control) or 25 mM BABA. (B) Percentage of *S. avenae* feeding on MilliQ water (H_2_O), standard artificial diet (control) and artificial diet containing 50 mM BABA (BABA). Shown are mean ± SEM (n = 14). Different letters indicate statistically significant differences (*P*<0.05, Turkey's HSD test).

BABA-treated wheat plants were not attractive or repellent to *S. avenae* (Fig. 2A). *Sitobion avenae* preferred artificial diets than MilliQ water, but showed no preference between standard artificial diet and artificial diet containing 50 mM BABA (Fig. 2B).

### Free amino acid composition of phloem sap

**Figure 3:**
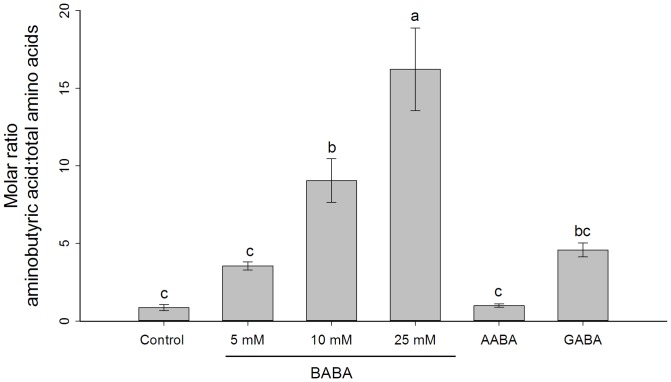
Relative aminobutyric acid levels in phloem sap of wheat seedlings. Plants were soil-drenched with different concentrations of BABA, 50 mM AABA and 50 mM GABA. Phloem sap was collected 3 days after treatment. Shown are mean ± SEM (n = 6). Different letters indicate statistically significant differences (*P*<0.05, Turkey's HSD test).

The relative concentrations of aminobutyric acid in phloem sap of BABA-treated wheat plants were higher than that in control plants (*F* = 20.13, df  = 3, 20, *P*<0.001; Fig. 3). Because wheat plants could produce GABA not BABA, we consider that the aminobutyric acid in control plants was GABA. Our method cannot discriminate BABA from its isomers, and the excess amount of aminobutyric acid in BABA-treated plants was assumed to be BABA. Soil drench- with AABA (*P* = 1) did not influence aminobutyric acids concentration in wheat phloem, while GABA (*P* = 0.313) only slightly increased aminobutyric acids levels.

Except for BABA, the relative concentration of serine in BABA-treated wheat phloem sap was also significantly less than that in control plants (*t* = 2.78, df  = 10, *P* = 0.02; Fig. S2), while other amino acids levels between treatments were similar (Fig. S2).

### Free amino acid composition of *Sitobion avenae*

**Figure 4:**
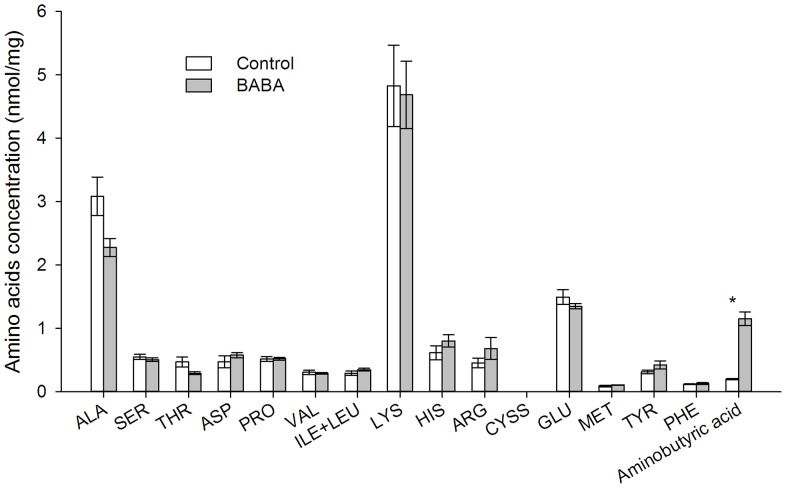
Amino acid concentration in *Sitobion avenae* body. Wheat plants were soil-drenched with MilliQ water (control) and 25 mM BABA. Newly-born nymphs feeding on control or BABA-treated plants for 7 days were collected for analysis. Shown are mean ± SEM (n = 4, *P<0.05, Student's *t*-test).

The relative concentration of aminobutyric acid in *S. avenae* feeding on BABA-treated plants was significantly higher than those feeding on control plants (*t* = 8.84, df  = 6, *P*<0.001; Fig. 4). The high levels of aminobutyric acid in *S. avenae* should be BABA from phloem sap of BABA-treated plants. The relative levels of alanine (*t* = 2.41, df  = 6, *P* = 0.052) and threonine (*t* = 2.17, df  = 6, *P* = 0.073) in aphids feeding on BABA-treated plants were numerically lower than those feeding on control plants (Fig. 4). The ratio of other amino acids was similar.

### Residual effect of BABA treatment

**Figure 5:**
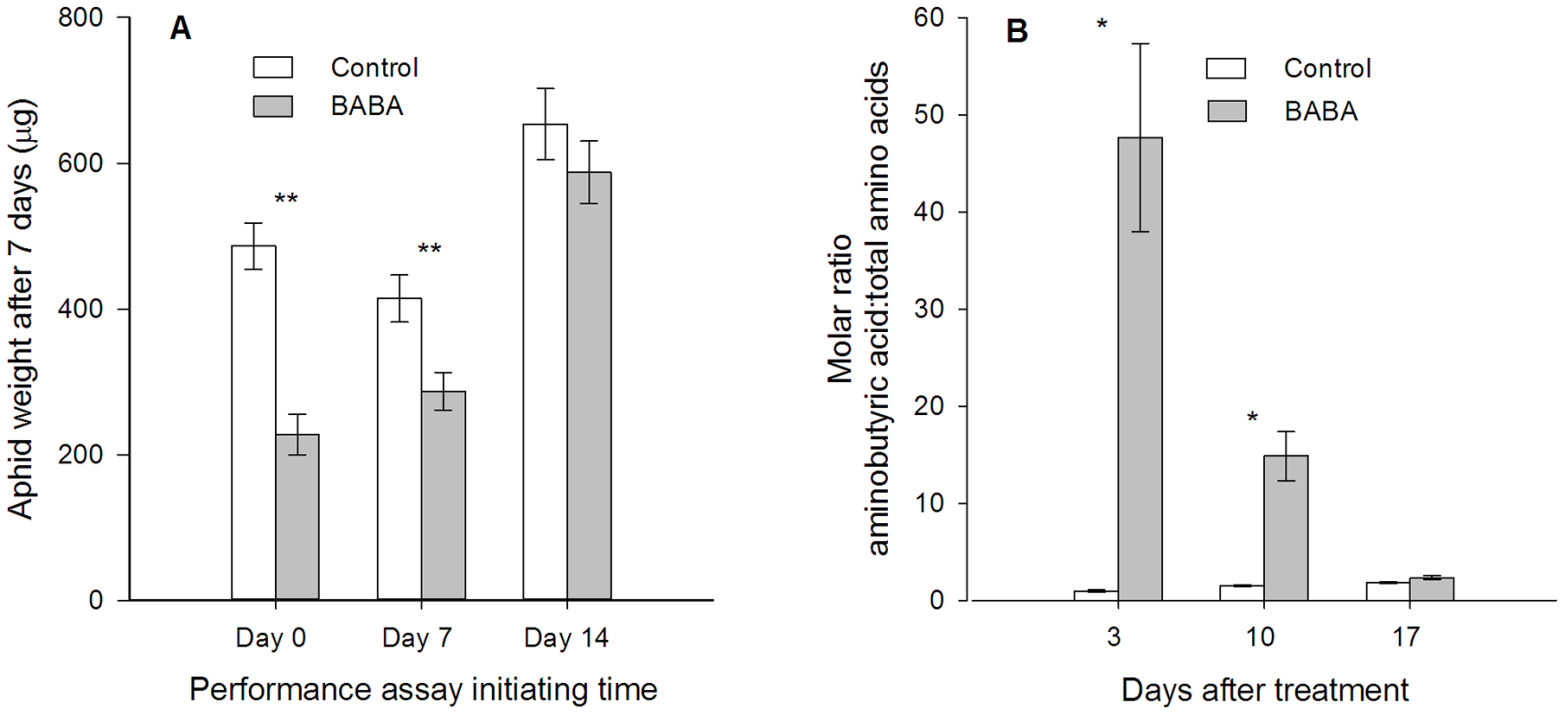
Effect of BABA treatment on *Sitibion avenae* performance and relative aminobutyric content in plant phloem. (A) Weights of *S. avenae* after feeding on control and BABA-treated plants for 7 days. Performance assay started 0, 7 and 14 day after treatment. (B) Relative aminobutyric acid levels in wheat seedlings phloem, which was collected 3, 10 and 17 days after treatment. Shown are mean ± SEM (*P<0.05, **P<0.01, Student's *t*-test).

After 7 days of BABA treatment, wheat seedlings still reduced *S. avenae* growth (*t* = 3.10, df  = 56, *P*<0.01; Fig. 5A), which was positively correlated with aminobutyric acid levels in plant phloem sap (Fig. 5B). The relative aminobutyric acid concentration in BABA-treated plants was higher than that in control plants 3 days (*t* = 4.827, df  = 6, *P* = 0.003) and 10 days (*t* = 5.286, df  = 6, *P* = 0.002) after treatment, but not significant after 17 days (*t* = 0.217, df  = 6, *P* = 0.073) (Fig. 5B).

### Feeding activities of *Sitobion avenae*

The feeding behavior of *S. avenae* on BABA-treated and control plants was similar based on the results of the EPG technique (Table 1). The number of probes (*P* = 0.376), total duration of probing (*P* = 0.982), total duration of salivation period (E1) (*P* = 0.808), and total duration of phloem feeding (E2) (*P* = 0.460) were not significantly different, suggesting that BABA treatment did not induce physical barriers or chemical deterrents in wheat seedlings against *S. avenae* (Table 1).

**Table 1:** EPG parameters of *Sitobion avenae* during an 8-h recording on wheat seedlings soil-drenched with water (Control) or *β*-aminobutyric acid (BABA) 48 h earlier.

EPG Parameters	Control n = 21	BABA n = 25
Number of probes	10.7±1.1	13.9±2.1
Number of short probes (<3 min)	4.0±0.6	5.5±1.2
Number of probes to the 1st E1	7.8±1.3	8.6±1.0
Time from 1st probe to 1st E1	2.3±0.4	2.6±0.3
Total duration of probing (h)	7.1±0.1	7.0±0.2
Total duration of pathway	2.1±0.2	2.3±0.2
Total duration of E2 (h)	4.1±0.3	3.8±0.4
Mean duration of E2 (h)	1.7±0.3	2.4±0.5
Total duration of E1 (min)	16.0±4.1	12.8±2.3
Mean duration of E1 (min)	5.9±1.6	5.2±1.2
Number of E2	3.0±0.3	2.6±0.4
Number of E1	3.1±0.4	2.9±0.4
Duration of xylem ingestion (h)	0.6±0.2	0.6±0.1

Values represent means (± SEM). E1: Salivation phase; E2: Phloem sap ingestion. n: number of replicates.

### POD activities

There were no difference for POD activities 2 days (*t* = 0.581, df  = 12, *P* = 0.572), 4 days (*t* = 0.860, df  = 7.13, *P* = 0.418) and 6 days (*t* = 1.914, df  = 12, *P* = 0.080) following treatment between control and BABA-treated plants (Fig. S3A). Also, the BABA treatment did not increase POD activities of aphid infested wheat seedlings compared with aphid infested control plants 2 days after treatment (*P* = 0.932) (Fig. S3B). Activities of POD in aphid infested wheat seedlings were higher than those in aphid infested plants previously treated with BABA (*P* = 0.032).

### Direct toxicity of BABA on *Sitobion avenae*

**Figure 6:**
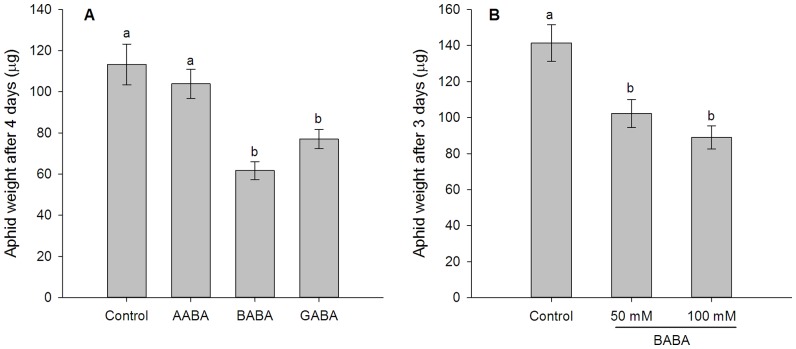
Direct toxicity of BABA to *Sitobion avenae*. (A) Weights of nymphs after feeding on standard artificial diet (control) and artificial diet containing 50 mM AABA, BABA or GABA for 4 days. (B) Weights of nymphs after feeding on standard artificial diet (control) and artificial diet containing 50 mM BABA or 100 mM BABA for 3 days. Shown are mean ± SEM. Different letters indicate statistically significant differences (*P*<0.05, Turkey's HSD test).

*Sitobion avenae* feeding for 4 days on artificial diets containing BABA or GABA had lower weights than those feeding on standard artificial diet; whereas those feeding on artificial diet containing AABA had no influence on aphid weights (*F* = 11.92, df  = 3, 35, *P*<0.001; Fig. 6A). *Sitobion avenae* feeding on artificial diet containing BABA had only 55% of the weights of those feeding on standard artificial diet (Fig. 6A). Also, *S. avenae* feeding for 3 days on standard artificial diet had higher weights than those feeding on artificial diets containing 50 mM or 100 mM BABA (*F* = 11.33, df  = 2, 49, *P*<0.001; Fig. 6B). The mortality of *S. avenae* feeding on all artificial diets was similar (*F* = 2.381, df  = 3, 36, *P* = 0.086; 22% for control, 20% for AABA, 46% for BABA, 42% for GABA).

## Discussion

Although numerous studies have examined the effects and mechanisms of BABA-mediated plant resistance to plant pathogens, few studies have investigated the role of BABA in plant resistance to insects [4], [9]. Previous researchfound that BABA application suppressed performance of some aphids (*A. pisum*, *M. persicae* and *B. brassicae*), caterpillars (*T. ni* and *P. xylostella*), and *D. citri* [10]–[12]. Our results demonstrated that BABA applied as a root drench significantly reduced weights of *S. avenae*, and the effects was dose dependent. We found high concentration of BABA in treated plant phloem sap, and the BABA level remained higher than that in control phloem sap after 10 days, suggesting that wheat could absorb BABA by roots and metabolize or decompose BABA slowly. The BABA-mediated suppression of aphid growth lasted at least for 7 days, which is correlated with BABA contents in phloem sap. It was assumed that resistance to insects induced by BABA is not based on its direct toxicity [10], [12]. In contrast, we found that *S. avenae* feeding on artificial diets containing BABA had reduced weights, suggesting that BABA had direct toxic effects on *S. avenae*.

Our results demonstrated that BABA reduced *S. avenae* growth on wheat only when drenched into the soil, and BABA-mediated wheat resistance to *S. avenae* was positively correlated with BABA concentration in wheat phloem sap. By using 14C-labled BABA, Cohen and Gisi (1994) found that only a small proportion of foliarly applied BABA is taken up by plant leaves [23]. Foliar spray and seed treatment with BABA may result in relatively lower BABA concentration in wheat phloem and thus have no effects on aphid performance. Root drench with BABA rendered wheat plants a durable resistance to *S. avenae*, which corresponds to high BABA concentration in wheat phloem.

*Sitobion avenae* could not discriminate the artificial diet containing BABA from the standard artificial diet, implying that *S. avenae* may not have corresponding receptors. Because BABA is rarely found in plants, aphids have few chances to encounter this compound in nature and do not evolve the ability to perceive BABA [24]. Moreover, we did not find any influence of BABA application on *S. avenae* host preference, indicating that BABA did not induce any repellents in wheat seedlings. Also the EPG data indicated that *S. avenae* exhibited similar feeding activities on control and BABA-treated plants, which is consistent with host preference results. These results suggest that neither BABA itself nor BABA treated wheat plants has antifeedant effects on *S. avenae*. Because *S. avenae* had similar phloem feeding duration on control and BABA-treated plants, the reduced performance of this aphid on BABA-treated wheat plants may not be due to limiting aphid feeding or a lack of nutrition.

BABA-induced plant resistance to pathogens is mainly through callose formation and SA, ABA signaling pathway [3], [9], [25]. BABA induced callose deposition occurs at the sites of pathogen penetration; thus preventing spread of the pathogen [3], [25]. Callose is also involved in plant phloem sealing mechanisms, which could confer plant resistance to aphids [26], [27]. Aphids inject watery saliva to prevent plugging and sealing of sieve plates [27]. This ejection of saliva can be detected by the EPG as E1 salivation. However, total or mean duration of E1 on control and BABA-treated plants was not significantly different. In addition, aphids had similar total as well as mean duration of phloem sap ingestion. Therefore, BABA-mediated wheat resistance to *S. avenae* is not possibly based on callose-mediated phloem occlusion. JA is the most important cellular signal in plant immunity to most insect herbivores [28]. Application of JA or its derivatives methyl jasmonate could increase plant secondary metabolites and resistance to a broad spectrum of insect herbivores [28], [29]. Our previous work showed that both methyl jasmonate and SA treatment deterred *S. avenae* preference and feeding activities, but had no effects on aphid performance [19]. These rule out the possible involvement of JA and SA signaling transduction pathways in the BABA-mediated wheat resistance to *S. avenae*.

It has been believed that BABA-mediated plant resistance to pathogens and insects is not based on its direct toxicity [10], [12], [24]. Recently, however, Šašek et al. (2012) found BABA had direct antifungal activity against *Leptosphaeria maculans* and the effect was comparable with the fungicide tebuconazole [30]. Our study showed that both BABA and GABA had direct toxicity to *S. avenae* when added to artificial diet. GABA is the major inhibitory neurotransmitter in vertebrate and invertebrate nervous systems [31], [32]. Synthetic diet containing GABA reduced growth and survival of oblique-banded leaf roller larvae [33]. GABA possibly reduced *S. avenae* performance by disturbing the aphid nervous system [32]. However, the mechanism of BABA direct suppression of aphid growth is still unknown. In contrast to our findings, Hodge et al. (2005) did not find direct toxicity of BABA to *A. pisum*, and Tiwari (2013) reported that BABA had no influence on *D. citri* survival [10], [12]. It is possible that their application methods only lead to low accumulation of BABA in insects, which was not high enough to reduce insects' fitness. Our data showed that the aminobutyric acid concentration in *S. avenae* feeding on wheat plant treated with BABA was 5.8 times higher than that on control plants, assuming the excess aminobutyric acid in *S. avenae* is BABA. Another possibility is that the direct toxicity of BABA to insects is species-specific.

BABA treatment can alter the amino acid balance in *Arabidopsis*, and l-glutamine inhibited BABA-induced resistance to thermotolerance and a bacterial pathogen [34], [35]. We found that BABA-treated wheat phloem accumulated less serine than water-treated control. These findings imply that BABA possibly involves in plant amino acid metabolism. *Sitobion avenae* feeding on BABA-treated wheat seedlings had slightly lower concentration of alanine (*P* = 0.052) in their bodies. The lower alanine concentration in *S. avenae* feeding on BABA-treated plants may not be due to difference in alanine concentrations in plant phloem, because BABA treatment did not decrease wheat phloem alanine content. These results suggest that BABA could also change aphid amino acid metabolism by an unknown mechanism. Furthermore, glycine is an important inhibitory neurotransmitter in the central nervous system, while BABA is a partial agonist at the glycine receptor [36]. Low concentrations of BABA competitively inhibits glycine responses, whereas higher concentrations elicit a significant membrane current [36]. Therefore, BABA probably exerted direct effects against *S. avenae* by causing aphid neurological disorders.

In this study, we have shown that BABA applied as a soil drench reduced aphid performance, while had no influence on aphid behavior. Our results suggest that BABA have direct toxicity to *S. avenae*. Therefore, the underlying mechanism of BABA-mediated plant resistance to phytopathogens and insect herbivores may be different. These findings expand our knowledge of BABA-mediated plant resistance to insects, but the precise mechanism of direct toxicity of BABA to insects remained unknown. Further research is also needed to investigate whether BABA has direct toxicity to other insects as well as non-target organisms.

## Supporting Information

Figure S1 **Fresh weights of wheat seedlings soil-drenched with different concentrations of BABA.** Shoot above root was harvested 7 days after treatment. Shown are mean ± SEM (n = 9–10). Different letters indicate statistically significant differences (*P*<0.05, Turkey's HSD test).
(TIF)

Figure S2 **Relative amino acids concentrations in phloem of wheat seedlings soil drenched with MilliQ water (control) and 25 mM BABA.** Phloem sap was collected after 3 days of treatment. Error bars represent SEM from 6 biological replicates. (* *P*<0.05, Student's t-test).
(TIF)

Figure S3 **Peroxidase (POD) activities of wheat leaves.** Wheat seedlings were soil-drenched with MilliQ water (control) and 25 mM BABA. (A) POD activities of control and BABA-treated plants. (B) POD activities in control plants, control plants infested with fifteen 3rd instar-adult *S. avenae*, BABA-treated plants, BABA-treated plants infested with fifteen 3rd instar-adult *S. avenae*. Shown are mean ± SEM. Different letters indicate statistically significant differences (*P*<0.05, Turkey's HSD test).
(TIF)

Table S1 **Composition of standard artificial diet for** ***Sitibion avenae*** **.**
(PDF)

Table S2 **Masses of precursor and product ions and collision energy for liquid chromatography-electrospray ionization tandem mass spectrometry (LC-ESI-MS-MS) analysis of amino acids.**
(PDF)
