# Supplementary figures and images for: Deciphering the Mechanism of β-Aminobutyric Acid-Induced Resistance in Wheat to the Grain Aphid, Sitobion avenae
Source: PLoS One. 2014 Mar 20;9(3):e91768. doi: 10.1371/journal.pone.0091768 (PMC3961263; doi:10.1371/journal.pone.0091768)

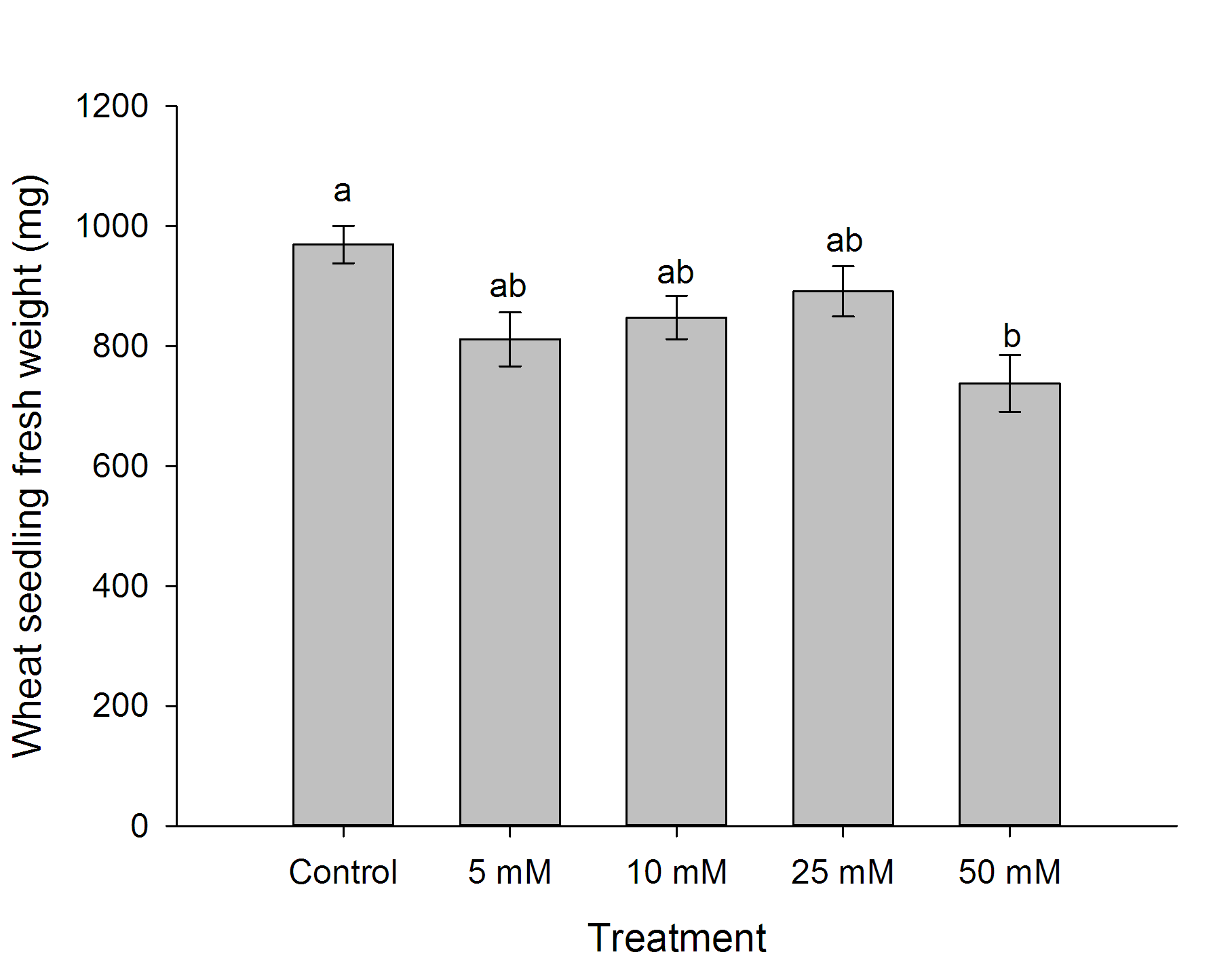

Supplement: Figure S1 — Fresh weights of wheat seedlings soil-drenched with different concentrations of BABA. Shoot above root was harvested 7 days after treatment. Shown are mean ± SEM (n = 9–10). Different letters indicate statistically significant differences (P<0.05, Turkey's HSD test). (TIF) [file pone.0091768.s001.tif]

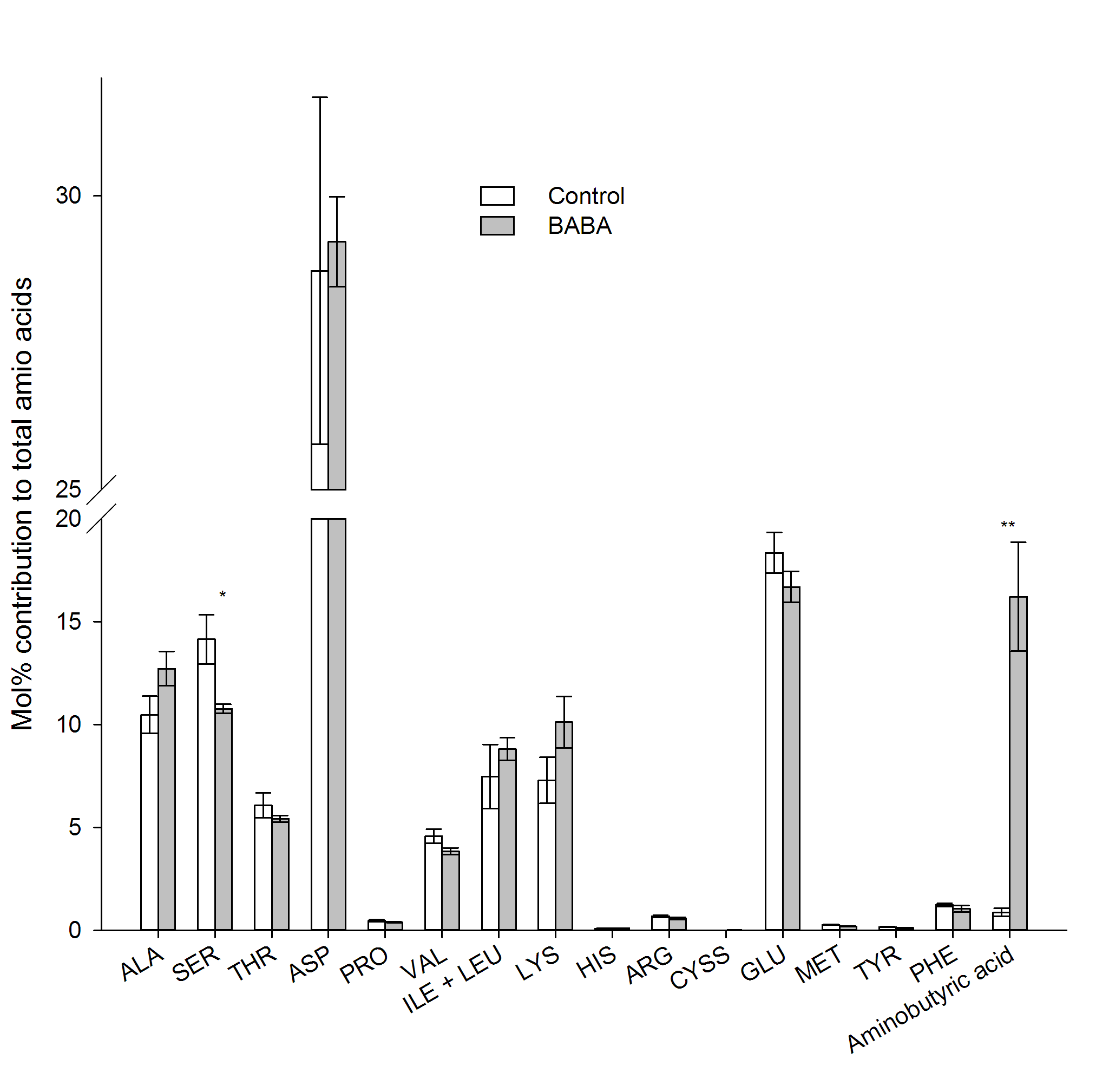

Supplement: Figure S2 — Relative amino acids concentrations in phloem of wheat seedlings soil drenched with MilliQ water (control) and 25 mM BABA. Phloem sap was collected after 3 days of treatment. Error bars represent SEM from 6 biological replicates. (* P<0.05, Student's t-test). (TIF) [file pone.0091768.s002.tif]

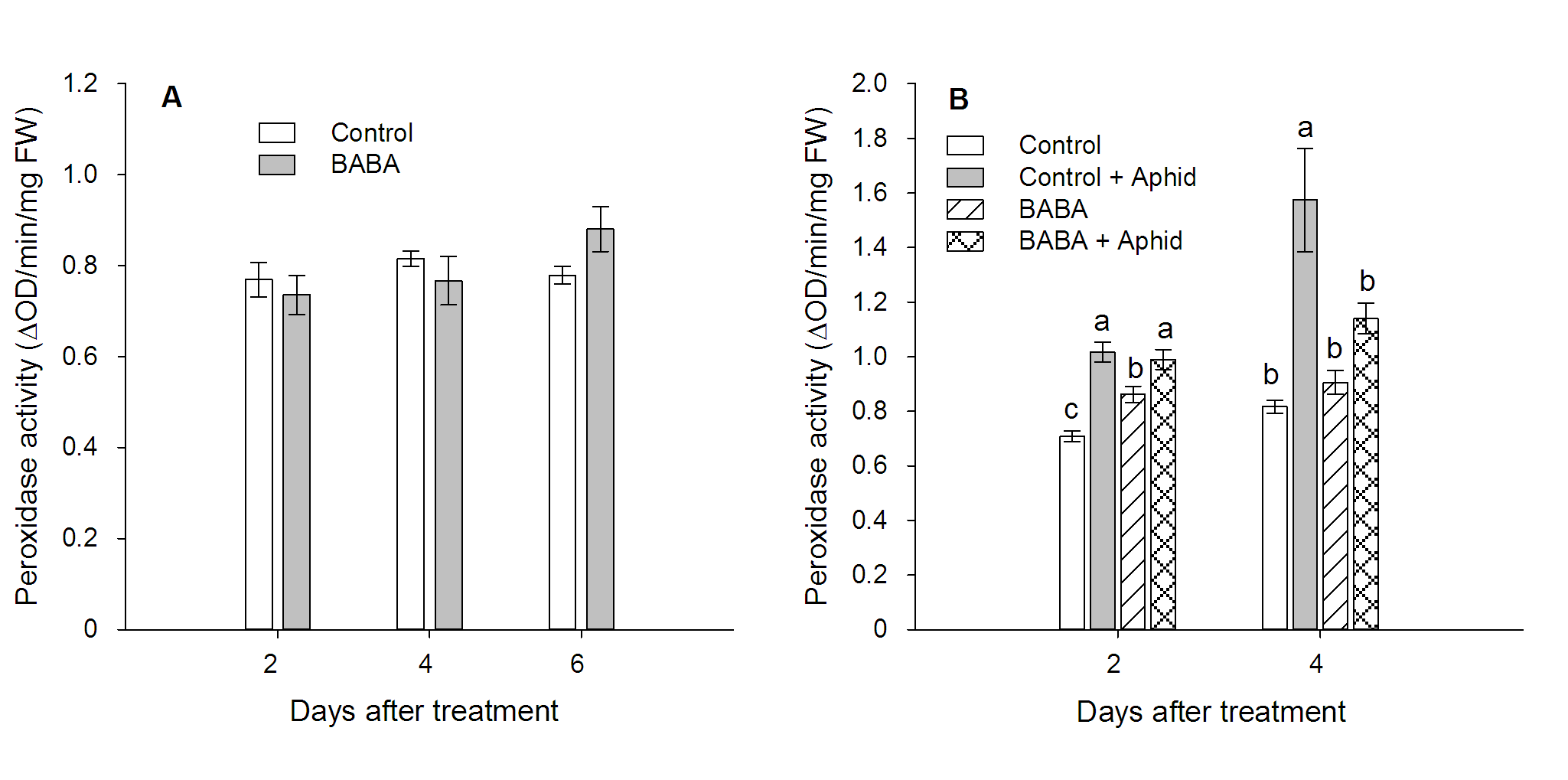

Supplement: Figure S3 — Peroxidase (POD) activities of wheat leaves. Wheat seedlings were soil-drenched with MilliQ water (control) and 25 mM BABA. (A) POD activities of control and BABA-treated plants. (B) POD activities in control plants, control plants infested with fifteen 3rd instar-adult S. avenae, BABA-treated plants, BABA-treated plants infested with fifteen 3rd instar-adult S. avenae. Shown are mean ± SEM. Different letters indicate statistically significant differences (P<0.05, Turkey's HSD test). (TIF) [file pone.0091768.s003.tif]
