# Supplementary material for: Deciphering the Mechanism of β-Aminobutyric Acid-Induced Resistance in Wheat to the Grain Aphid, Sitobion avenae
Source: PLoS One. 2014 Mar 20;9(3):e91768. doi: 10.1371/journal.pone.0091768 (PMC3961263; doi:10.1371/journal.pone.0091768)
Supplement: Table S1 — Composition of standard artificial diet for Sitibion avenae . (PDF) [file pone.0091768.s004.pdf]

Table S1 Composition of standard artificial diet for *Sitobion avenae*.

L-Amino acids and amides (mg)		***Others***	
Alanine	30	NaCl	1.271 mg
Arginine	400	$CuCl_2•2H_2O$	0.3 mg
Asparagine	200	$FeCl_3•6H_2O$	2.2 mg
Aspartic acid	69	$MnCl_2•4H_2O$	0.79 mg
Cysteine	50	$ZnCl_2$	0.471 mg
Cystine	1	$MgCl_2•6H_2O$	150 mg
Glutamic acid	200	KH_2PO_4	200 mg
Glutamine	600	Cholesterol	5 mg
Glycine	20	Sucrose	15.4 g
Histidine	200	Water to make	100 mL
DL-Homoserine	50		
Isoleucine	100		
Leucine	42.5		
Lysine mono-HCl	200		
Methionine	100		
Phenylalanine	100		
Proline	100		
Serine	100		
Threonine	200		
Tryptophan	150		
Tyrosine	20		
Valine	37.2		
Vitamins (mg)			
Ascorbic acid	100		
Biotin	0.1		
Calcium pantothenate	5		
Choline chloride	50		
Folic acid	1		
Inositol	50		
Nicotinic acid	10		
p-Aminobenzoic acid	10		
Pyridoxine HCl	2.5		
Riboflavin	0.5		
Thiamine HCl	2.5		
