# Supplementary material for: Deciphering the Mechanism of β-Aminobutyric Acid-Induced Resistance in Wheat to the Grain Aphid, Sitobion avenae
Source: PLoS One. 2014 Mar 20;9(3):e91768. doi: 10.1371/journal.pone.0091768 (PMC3961263; doi:10.1371/journal.pone.0091768)
Supplement: Table S2 — Masses of precursor and product ions and collision energy for liquid chromatography-electrospray ionization tandem mass spectrometry (LC-ESI-MS-MS) analysis of amino acids. (PDF) [file pone.0091768.s005.pdf]

Table S2 Masses of precursor and product ions and collision energy for liquid chromatography-electrospray ionization tandem mass spectrometry (LC-ESI-MS-MS) analysis of amino acids.

Amino acid	Precursor ion $[M+H]^+$ (*m/z*)	Product ion (*m/z*)	Collision energy (eV)
Ala	90	70	0
Ser	106	60	35
Pro	116	70	35
Val	118	72	35
Thr	120	74	35
Cyss	241	152	35
Ile/Leu	132	86	35
Asp	134	74	35
Lys	147	130	35
Glu	148	130	35
Met	150	133	35
His	156	110	35
Phe	166	120	35
Arg	175	116	35
Tyr	182	136	35
Aminobutyric acid	104	86	35
